# Smartphone-driven centrifugal microfluidics for diagnostics in resource limited settings

**DOI:** 10.1007/s10544-024-00726-x

**Published:** 2024-10-26

**Authors:** Noa Lapins, Ahmad S. Akhtar, Indradumna Banerjee, Amin Kazemzadeh, Inês F. Pinto, Aman Russom

**Affiliations:** 1 Division of Nanobiotechnology, Department of Protein Science, Science for Life Laboratory, KTH Royal Institute of Technology, Solna, Sweden; 2 AIMES – Center for the Advancement of Integrated Medical and Engineering Sciences at Karolinska Institutet and KTH Royal Institute of Technology, Stockholm, Sweden

**Keywords:** Centrifugal microfluidics, Colorimetry, Point-of-care diagnostics, Resource limited settings

## Abstract

The broad availability of smartphones has provided new opportunities to develop less expensive, portable, and integrated point-of-care (POC) platforms. Here, a platform that consists of three main components is introduced: a portable housing, a centrifugal microfluidic disc, and a mobile phone. The mobile phone supplies the electrical power and serves as an analysing system. The low-cost housing made from cardboard serves as a platform to conduct tests. The electrical energy stored in mobile phones was demonstrated to be adequate for spinning a centrifugal disc up to 3000 revolutions per minute (RPM), a rotation speed suitable for majority of centrifugal microfluidics-based assays. For controlling the rotational speed, a combination of magnetic and acoustic tachometry using embedded sensors of the mobile phone was used. Experimentally, the smartphone-based tachometry was proven to be comparable with a standard laser-based tachometer. As a proof of concept, two applications were demonstrated using the portable platform: a colorimetric sandwich immunoassay to detect interleukin-2 (IL-2) having a limit of detection (LOD) of 65.17 ng/mL and a fully automated measurement of hematocrit level integrating blood-plasma separation, imaging, and image analysis that takes less than 5 mins to complete. The low-cost platform weighing less than 150 g and operated by a mobile phone has the potential to meet the REASSURED criteria for advanced diagnostics in resource limited settings.

**Supplementary Information:**

The online version contains supplementary material available at 10.1007/s10544-024-00726-x.

## Introduction

For any healthcare system, centralized laboratories are the mainstay where most of the standard diagnostic procedures are performed. These laboratories and standard procedures require skilled technicians and costly equipment. Moreover, the diagnostic results in these laboratories can take from several hours to days which may result in a delay between sampling and treatment and/or increase in the cost (Suea-Ngam et al. 2020). These problems get further intensified in resource limited settings (RLS) where the healthcare related services are expensive, for majority of the population, and often only available in big cities with access to centralized labs. This disparity became evident during the COVID-19 pandemic, as it was observed that the countries which managed to scale up their testing capability were able to identify and isolate infected patients more effectively (Harpaldas et al. 2021). On the other hand, low-income countries that do not have the infrastructure to ramp-up their testing (Soares et al. 2021) were left to choose between the option to live with the virus and achieve herd immunity or implement wide scale lockdown measures (Kalk and Schultz 2020). To make sensitive and specific diagnostic tests accessible to every individual, innovative approaches are needed that do not rely on expensive and bulky equipment.

A point of care (POC) device performs diagnostics at or near the patient side and results in improved patient management (Chen et al. 2019; Wang et al. 2021). In general, a POC device should fulfil the ASSURED criteria (Mabey et al. 2004) coined by WHO in 2003, which stands for affordable, sensitive, specific, user-friendly, rapid and robust, equipment free or little equipment and deliverable to end-user (Mabey et al. 2004; Tay et al. 2018; Manoto et al. 2018). The ASSURED criteria was updated to REASSURED in 2019 by Peeling et al*.*, adding real time connectivity and ease of sample collection, owing to recent developments in digital technology (Land et al. 2018). Similar to the criteria for POC tests, the solutions have also gone through an evolution. The most common and earliest example of POC device is the lateral flow tests which are cheap, easy to use and provide rapid results (Tsai et al. 2019; Iles et al. 2022). They have been used extensively for a wide range of diseases such as hepatitis, syphilis, human immunodeficiency virus (HIV) and malaria (Jani and Peter 2013). However, these tests are not a generic solution and suffer from challenges such as lack of quantitative results, reproducibility, sensitivity, and specificity (Carrell et al. 2019; Ince and Sezgintürk 2022). The next generation of POC devices consisted of test cartridges which are processed in benchtop devices. Such POC devices use the advancements in microfluidics and microelectronics to perform more complicated diagnostic assays such as nucleic acid amplification tests (NAATs) and multiplexed immunoassays along with quantitative measurement for viral load or the biomarkers of interest (Jani and Peter 2013). However, these benchtop devices have a high initial cost with the recurrent cost of test cartridges and require continuous provision of electricity, which makes them more suitable to be used in hospitals or smaller clinics but limits their application in resource limited settings (Garrett et al. 2016; Dorward et al. 2018). Following the COVID-19 pandemic, the trend has been towards an integrated solution which employs microfluidics to perform complex assays using simple and smart connected systems for data transmission to key stakeholders (Harpaldas et al. 2021).

In this context, centrifugal microfluidics based lab on a disc devices provide an attractive solution as it needs minimal equipment for driving and manipulating fluids without any external tubing or pump (Wang et al. 2021). These devices are capable of integration and automation of multiple steps of complex diagnostic assays using only the centrifugal force modulated by the rotational speed. Compared to a lateral flow test, they can be used to perform more complex assays while keeping the cost of the device lower compared to a commercial benchtop device using test cartridges. To date, several publications have reported different applications of lab on a disc device in diagnostics, food poisoning, forensics, etc. (Soares et al. 2021; Akhtar et al. 2023; Klatt et al. 2021; Oh et al. 2016; Choi et al. 2016; Teixeira et al. 2022; Van Nguyen and Seo 2021; Woolf et al. 2023). Examples of both flow control mechanisms and assays conducted on these platforms can be found in several research and review papers (Strohmeier et al. 2015; Kazemzadeh et al. 2015; Shi et al. 2021; Miyazaki et al. 2020; Gilmore et al. 2016; Mishra et al. 2018; Nguyen et al. 2019). However, in order to bridge the gap between standard diagnostic methods and POC diagnostic in RLS, lab on a disc platforms need to address certain challenges such as lack of continuous and reliable electricity to drive the motor and/or to operate PC-based user interface (Gilmore et al. 2016; Smith et al. 2016).

The combination of smartphones and POC diagnostics, also known as mobile health (mHealth) platforms (Wang et al. 2023), can provide an alternative to bulky imaging and analysis systems and has the potential to deliver diagnostic testing capability in RLS (Hernández-Neuta et al. 2019; Vashist and Luong 2019; Xu et al. 2018Dutta 2019). This combination has generally been focused on the use of smartphones as a portable imaging device using the camera module and/or employing the processing power of the smartphone for colorimetric or fluorescence readout (Kong et al. 2017; Yin et al. 2021; Nguyen et al. 2022; Yu et al. 2021; Draz et al. 2018; Sun et al. 2020; Jankelow et al. 2022; Wang et al. 2024). Another application has been to use the mobile phone as a graphical user interface through an app to control a POC device (Panpradist et al. 2021; García-Bernalt Diego et al. 2022; Lee et al. 2023). Delaney et al*.* connected the audio jack of a mobile phone to a pair of electrodes and by playing an audio file, induced an electrochemical reaction (Delaney and Hogan 2015). Thompson et al. used a mobile phone for analysing images to measure hematocrit level in whole blood (Thompson et al. 2016). Bhupathi et al*.* presented a mobile phone powered centrifuge that is used for sample preparation for LAMP assay (Bhupathi and Devarapu 2022). However, in all these examples mobile phones are being used to replace only one part of the diagnostic tests whereas they have the capability to replace multiple parts at the same time.

To make full use of a mobile phone as a multi modular accessory to a diagnostic platform, we developed a device that uses multiple functionalities of a mobile phone to conduct diagnostic tests. In this study, a combination of mobile phone and centrifugal microfluidics is introduced to demonstrate a fully integrated, low-cost, and portable POC diagnostic platform. The platform consists of a rotor, a potentiometer for speed control and a lens for imaging which are enclosed in a housing made from cardboard and powered by a mobile phone. The lightweight and portable housing is robust enough to carry out multiple POC tests. The mobile phone supplies the power required for spinning the centrifugal microfluidic disc and captures the images for further analysis. The embedded sensors in the phone are used to measure the rotational speed and provide feedback to the user, thus allowing speed control without the need for any additional sensors or electronic circuitry. As a proof of concept, a centrifugal microfluidic disc was designed to carry out a colorimetric sandwich immunoassay for detecting interleukin-2 (IL-2). In addition to that, a disc was designed to measure the hematocrit level in different blood samples using a simple metering and sedimentation technique. The experiments demonstrate the stability and versatility of the developed platform where the cardboard housing, speed control and the image analysis by mobile phone can be extended to a wide variety of applications for POC diagnostics in resource limited settings.

## Methods

### Platform fabrication and assembly

**Fig. 1:**
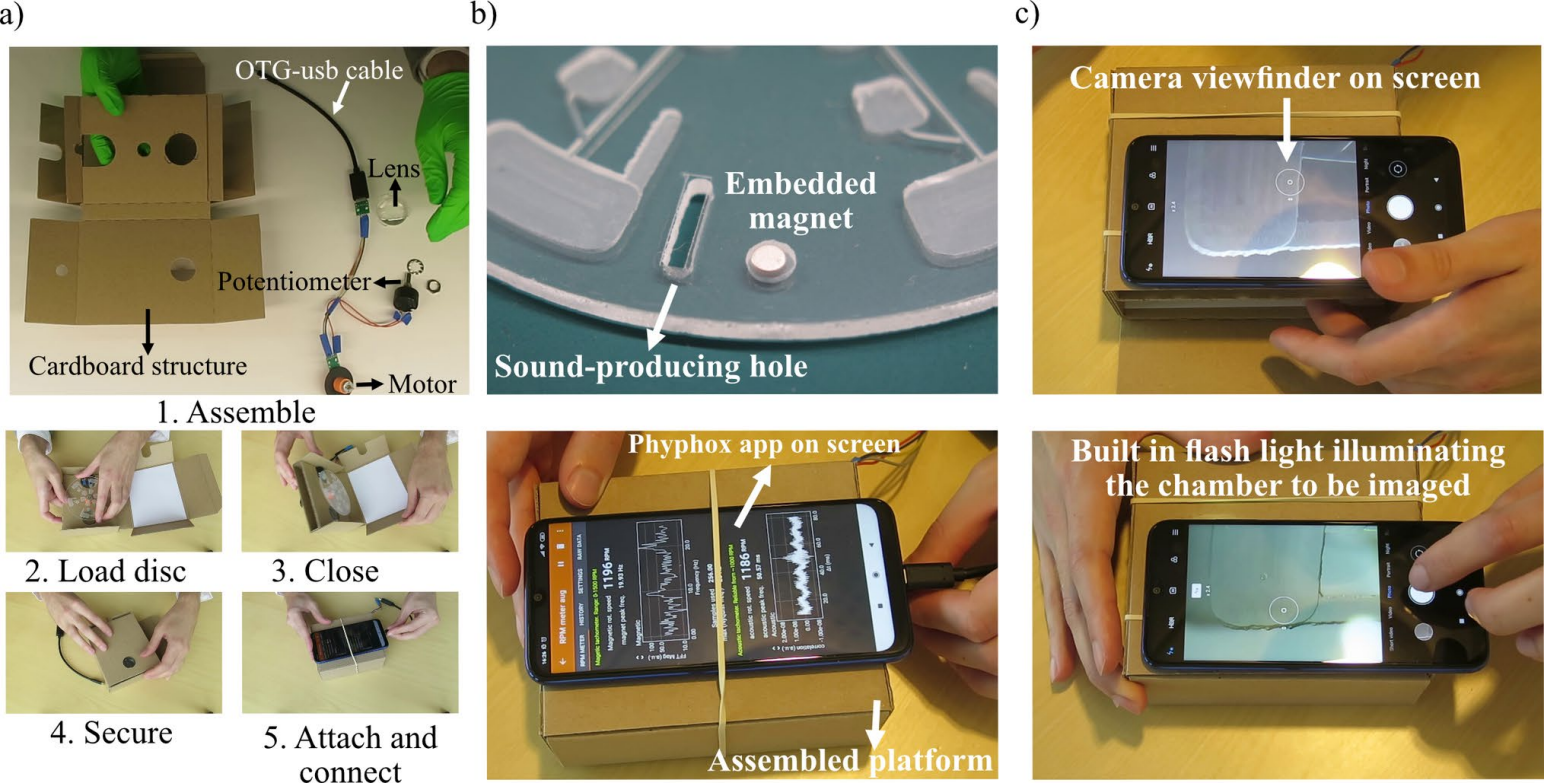
Smartphone-driven portable centrifugal microfluidic platform. **a**) The centrifugal platform in unassembled form showing all the components (1) the step-by-step assembly process (2–5) of the platform. **b**) Features added onto the disc to provide feedback to the user about the rotational speed of the disc using the in-built sensors of the phone (top). Signal generated by the features as seen by the user on the mobile phone app “phyphox” (bottom). **c**) Using the mobile phone to image channels and/or chambers inside the disc

To develop a POC device which is extremely low-cost and suitable to be used in resource limited settings, a housing structure made of cardboard was designed. Figure 1(a) shows the different components of the centrifugal platform and the assembly instructions for using the platform. The platform consists of the cardboard housing, a small DC motor, a potentiometer (RS PRO 250Ω Rotary Potentiometer; Article number 842–7080) for speed control, lens for imaging and a USB On-The-Go (OTG) cable for connection with the mobile phone. The cardboard housing is designed in a way that it can be assembled on site or in the field following some simple steps (Fig. S1) without any additional instruments. The housing is made from a single piece of cardboard. Folding lines and holes in the cardboard were made using a Graphtec CE6000-40 cutter plotter. Figure 1(b) shows the features added onto the disc for acoustic and magnetic tachometry used for speed control of the disc using the in-built sensors of the phone. These features combined with the in-built sensors of the phone provide feedback to the user regarding rotational speed of the disc which can then be controlled using a potentiometer. Figure 1(c) shows how a smartphone can be used for imaging of channels or chambers on the disc using the lens embedded in the cardboard housing. A flap on one side of the housing provides access to the disc while the disc is on the rotor. Circuit schematic for connecting the mobile phone to power and control the motor is shown in Fig. S2. USB On-The-Go is a standardized specification that allows other USB devices to be connected to a mobile phone (Doeven et al. 2015). In this case the mobile phone acts as the host and provides power to the peripheral device (motor).

### Disc fabrication

**Fig. 2:**
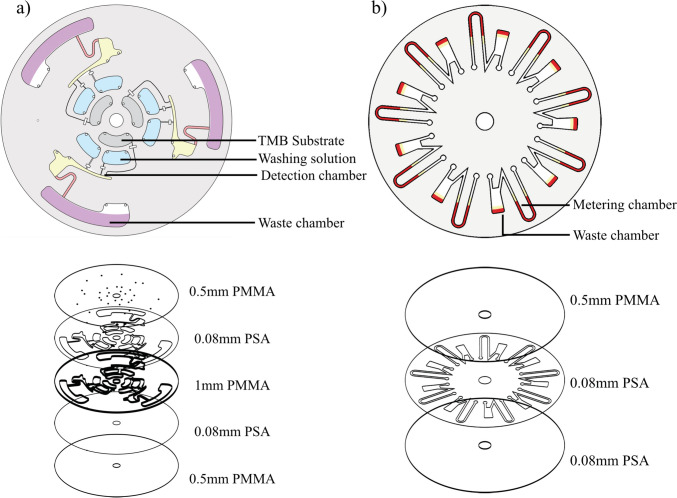
Exploded and assembled view of centrifugal microfluidic discs designed for conducting **a**) IL-2 ELISA and **b**) hematocrit level test

The discs were designed using the student version of AutoCAD® and cut using Roland MDX-40A milling machine. A more detailed description of the disc design can be found in section S1 and Fig. S3. The disc layers were bonded together using clear medical grade pressure-sensitive adhesives (ARCare® 90445 and 92712). The microstructures on pressure-sensitive adhesives were cut using a Graphtec CE6000-40 cutter plotter. After aligning the corresponding PMMA layers with the pressure-sensitive adhesive layer, the discs were subjected to a constant pressure for few hours in a manual press to complete the bonding. More details on fabrication of the centrifugal microfluidic discs can be found in our previous publications (Akhtar et al. 2023; Soares et al. 2021). Figure 2(a) shows the exploded view of a five-layered disc that was used for interleukin-2 (IL-2) sandwich ELISA. The disc was designed to integrate and automate multiple steps of ELISA using a combination of capillary valves and siphon valve. Three inlet chambers are used to load and store washing solution and TMB substrate on the disc at the start of the assay. The sequential release of these solutions is controlled by capillary valves which have different burst frequencies and can be controlled by changing the rotational speed of the disc. The three inlet chambers empty into the detection chamber which is used for incubation of sample solution to start the assay and colorimetric signal generation at the last step. The detection chamber is connected to the waste chamber through a siphon valve and as the level of liquid in the siphon goes past the crest of the siphon, it pulls all the liquid along with it to the waster chamber Fig. 2(b) shows a three-layered disc that was used for measuring the level of hematocrit in whole blood. It consists of a U-shaped metering channel that is connected to an overflow chamber. The U-shaped metering channel was designed to meter a specific volume of liquid (7.5 µL) when the disc rotates and to direct any excess liquid into the overflow chamber, similar to the design by Thompson et al. (Thompson et al. 2016).

### Sandwich ELISA on 96-well plate

The 96-well plate (Corning® 96-well Clear Flat Bottom Polystyrene, Cat. No. 3595) was coated with 100 μL of IL-2 antibody (1.04 mg/ml, ThermoFisher Scientific, Cat. No. P600) diluted to 20 μg/mL in PBS (Sigma Aldrich, Cat. No. P4417) and stored overnight at 4 ºC. The wells were then blocked with 100 μL of 5% (w/v) BSA (VWR, Cat. No. 0332) in PBS for 1 h at room temperature. Standards for calibration curve of IL-2 were prepared by diluting recombinant human IL-2 (ThermoFisher Scientific, Cat. No. PHC0026) in PBS to obtain the desired range of concentrations. 100 μL of the standard was added to the wells and incubated for 2 h at room temperature. For detection of bound IL-2, 100 μL of anti-IL2 (HRP) (1 mg/mL, Abcam, Cat. No. 106016) diluted to 5 μg/mL in PBS was added to the wells followed by addition of 100 μL of substrate (3, 3′,5,5′-Tetramethylbenzidine Liquid Substrate, Sigma Aldrich, Cat. No. T444). The reaction was stopped after 3 min by addition of 100 μL of 2 M H2SO4 and the absorbance was measured at 450 nm using a spectrophotometer (SpectraMax M5, Molecular Devices). Each step was followed by three washing steps using 0.05% Tween-20 (Merck, Cat. No. 822184) in PBS except after incubation with anti-IL2 (HRP) which was followed by five washing steps.

### Sandwich ELISA on centrifugal microfluidic platform

Detection chamber was coated with 100 μL of IL-2 antibody solution diluted to 20 μg/mL in PBS and stored overnight at 4 ºC. Blocking of the detection chamber was achieved by adding 100 μL of 4% (w/v) BSA in PBS and incubating for 30 min at room temperature. Sample solutions were prepared by spiking PBS with recombinant human IL-2 and mixing that with the detection antibody i.e., anti-IL2 (HRP) diluted to 5 μg/mL. To remove the unbound complexes, detection chamber was washed twice with 100 μL of 0.05% Tween-20 in PBS. At the last step, 70 μL of substrate was added into the detection chamber and the signal was allowed to develop for 3 min. For quantitative analysis, the final volume in the detection chamber was collected from the detection chamber and added to 70 μL of stop solution (2 M H_2_SO_4_) in a 96-well plate and absorbance at 450 nm was measured.

### Integration of ELISA steps on centrifugal microfluidic disc

As the first step, the disc was rotated at 300 RPM to activate the siphon and remove the capture antibody solution from the detection chamber. 4% BSA blocking solution was added, incubated for 30 min at room temperature and flushed out to the waste chamber through the siphon channel at 300 RPM. The sample and detection antibody solution prepared off-platform was loaded into the detection chamber and incubated for 30 min at room temperature. The washing solution and the TMB substrate were loaded onto the disc at the same time as the sample and detection antibody solution. The detection chamber was then emptied using the siphon channel at 300 RPM. The detection chamber was washed using the prestored washing buffer in washing chambers at 700 RPM and 900 RPM. For signal development, the TMB substrate was added into the detection chamber at 1400 RPM and the signal was allowed to develop on the disc before being transferred to a 96-well plate for quantification.

### Hematocrit level test

To ensure that metering is initiated during rotation, an unspecific volume of blood (~ 9 µL) was loaded using a pipette to completely fill the metering channel and partially fill the overflow chamber. To find the duration of centrifugation that is sufficient for an accurate analysis, triplicates of one blood sample were loaded in the way described above and analysed at different time intervals. All the hematocrit level tests were carried out from human blood samples stored in EDTA-treated tubes and purchased from Blodcentralen in Stockholm.

### Image processing for hematocrit test

An image analysis script was transcribed that uses an image processing toolbox in MATLAB (MathWorks, USA) to convert the original images to grayscale and correct the background stray marks. For the hematocrit level tests, first the backgrounds of the grayscale images are corrected and then the images are flattened using a Savitzky Golay filter in MATLAB. This increases the signal to noise ratio, with minimum distortion of the input signal. A histogram of the image is then generated, and a threshold level is defined. This threshold value distinguishes the hematocrit from the plasma in a single image i.e., hematocrit appears darker. Therefore, the sections in an image that have a grayscale value less than the threshold value correspond to the hematocrit concentration in blood.

### Speed control

To implement speed control for the rotational speed of the disc on the platform without the use of any external circuitry, the in-built sensors of the phone were employed combined with specific features embedded on to the disc which generate a signal that is recorded and measured using the mobile phone. To be able to measure and record data using the sensors of the mobile phone, an open-source app called phyphox (acronym for physical phone experiments) was used which is freely available on Google Play Store and Apple store (Staacks et al. 2018; Stampfer et al. 2020). Phyphox allows a user to access and record data from various integrated sensors of a phone and process it in real-time. Two different methods to measure the rotational speed of the disc were used.

#### Magnetic tachometer

A magnetic tachometer was implemented using the phyphox app to record and perform calculations on data from the internal magnetometer sensor of the phone. To use this feature for speed control, a small neodymium magnet (Diameter 5 mm; RS PRO Article # 219–2248) was embedded on one end of the disc as shown in Fig. 1(b). As the disc is rotated and the magnet passes below the phone’s magnetometer, a frequency spectrum is created from the magnetic field and the peak frequency is displayed on the screen which corresponds to the rotational frequency of the disc.

#### Acoustic tachometer

An acoustic tachometer was implemented using the phyphox app to record and perform calculations on data from the audio signal gathered from the phone’s internal microphone. To use this feature for speed control, a small sound generating hole was introduced in one end of the disc as shown in Fig. 1(b). As the disc is rotated, it generates a sound at a particular frequency that corresponds to the rotational frequency of the disc which is determined by calculating the autocorrelation of the recorded frequency spectrum.

#### Comparison with laser-based tachometry

The performance of the smartphone-based magnetic and acoustic tachometer was compared with a standard laser-based tachometer which is part of a stroboscope setup (Shimpo DT-311A Digital Stroboscope, Shimpo Instruments, USA). The setup uses a laser which shines downwards on to the surface of the disc and a reflective tape is placed on one part of the disc which reflects the laser back where a sensor detects this signal with every rotation and calculates the rotational speed of the disc. The disc was rotated at different rotational speeds in the range of 0 to 3000 RPM and the rotational speed was measured and compared.

## Results and discussion

### Smartphone powered centrifugal microfluidic platform

The centrifugal microfluidic platform was designed and developed exploiting one of the most inexpensive, accessible, and recyclable materials resulting in a unique and portable system appropriate for conducting POC tests at resource limited settings. The platform uses a light-weight housing made of cardboard that securely encloses a rotor, a lens, and a potentiometer for speed control. Using cardboard housing for the platform makes it a very environment-friendly solution for point of care testing where the platform itself can be reused or recycled while all the components of the system can be recovered and used subsequently in a newly assembled housing. Cardboard is one of the most common materials used for packaging and is easily available everywhere. The folding lines and cuts made in the cardboard facilitate the end user to assemble it in the field within a few minutes (Supplementary video S1). The total weight of the platform excluding the mobile phone is approximately 120 g. It uses the battery of the mobile phone for supplying electricity to spin the rotor and the camera of the mobile phone for monitoring and processing the assay results. The use of mobile phone for image and data processing has been thoroughly investigated in previously published research articles (Delaney and Hogan 2015; Lillehoj et al. 2013).

To keep the cost of the platform as low as possible, the in-built sensors of the phone were used to measure and provide feedback regarding the rotational speed of the disc. In addition to commonly used sensor such as the microphone, smartphones are equipped with a wide array of sensors, such as accelerometer, gyroscope, magnetometer etc. which perform different functions for the normal working of the phone. Phyphox is an open-source app that allows the user to obtain raw data of these sensors which can then be used for measurement of some physical quantities in real world scenario (Staacks et al. 2018; Stampfer et al. 2020). In centrifugal microfluidics, the fluidic operations are controlled by changing the rotational speed of the disc which makes the measurement of the rotational speed of the disc critical for carrying out any assay involving multiple steps. Generally, this would require the use of specialized sensors combined with external circuitry which would, in turn, increase the cost and footprint of the system.

As shown in Fig. 1(b), a simple rubber band can be used to secure the phone on top of the platform keeping the phone’s position fixed during the experiment, while the weight of the phone provides stability to the platform as the disc is rotating. After complete assembly of the platform with the disc mounted on the motor, the enclosure becomes dark making it difficult to image using the camera of the mobile phone. To overcome this problem, the camera flash of the mobile phone was used to illuminate the chambers and/or channel of the disc inside as shown in Fig. 1(c). A flap on the side of the platform can be used to access and rotate the disc manually for imaging (Supplementary video S2).

### Speed control

**Fig. 3:**
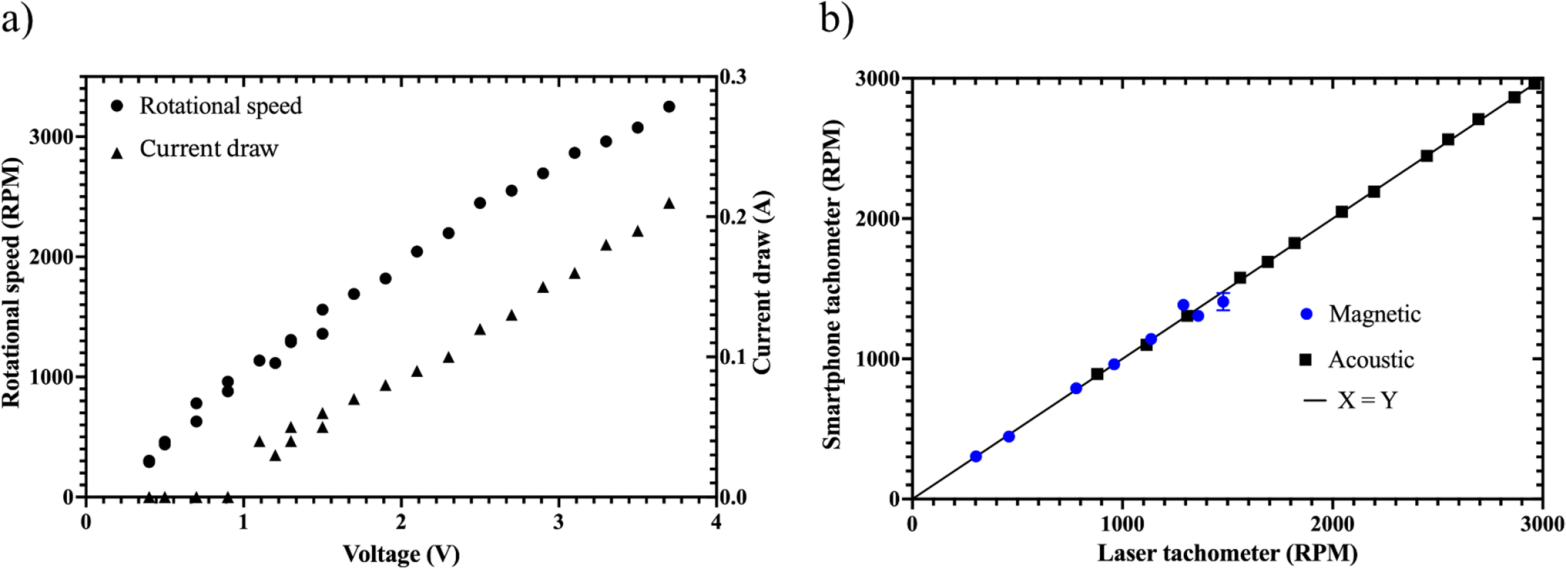
Speed control on the centrifugal microfluidic platform. **a**) Graph showing the relationship between voltage, current draw, and the rotational speed. **b**) Comparison between smartphone-based tachometer (magnetic and acoustic) and a standard laser-based tachometer. The error bars correspond to standard deviation of two measurements

As described above, to develop generic centrifugal microfluidics-based bioassays, it is crucial to control the rotational speed. Initially, the voltage requirement was characterized by the mobile phone to control the rotational speed. Figure 3(a) shows the relationship between the rotational speed and current draw of the motor as a function of the voltage supplied by mobile phone. The maximum voltage output from a phone’s charging port is 5 V. However, as soon as a motor is connected to the terminals, the voltage drops to 4.5 V due to voltage drop across the internal resistance of the battery. When a load, such as the motor, is connected to a power supply, it acts as a resistor in series and the current drawn from the supply is increased. This increase in current causes a voltage drop across the internal resistor of the battery which in turn decreases the voltage output. The rotor used in this case was recovered from a handheld USB fan which costs around $2. The low-cost rotor is capable of spinning the centrifugal disc, which has a diameter of 90 mm and thickness of 1.6 mm, at ~ 900 RPM at ~ 1 V. Figure 3(a) shows that a maximum rotational speed of 3250 RPM was achieved using the setup, which is higher than the rotational speeds generally used in centrifugal microfluidics (Akhtar et al. 2023; Okamoto and Ukita 2018; Wang et al. 2017; Gao et al. 2018; Malic et al. 2022; Ji et al. 2020; Dignan et al. 2021).

As discussed in Section 3.1, speed control of the centrifugal microfluidic disc is a requirement for integrating multiple steps of an assay as the flow and valving of liquid is controlled by the rotational speed. To achieve this, a combination of a potentiometer to control the speed and the embedded sensors in the phone to measure and display the rotational speed of the disc on the screen of the phone was used resulting in a closed loop feedback system. The potentiometer allows the user to control the voltage being supplied to the motor and hence, control the rotational speed of the disc.

Section 2.8 introduced the strategy for the measurement of rotational speed using the sensors of the phone and an open-source app called phyphox. Two different customized “experiments” were designed in the “phyphox editor” for the purpose of measuring rotational speed. As shown in Fig. 1(b), a sound producing hole was added to the disc to generate a sound at a frequency that corresponds to its rotational speed. The acoustic tachometer program was designed to capture 100 ms of audio samples at a signal acquisition rate of 48000 Hz and then calculate its autocorrelation (Fig. S4). By doing this, the frequency and time period of a single frequency audio signal is determined by autocorrelation of the audio signal being picked up by the microphone of the mobile phone. The noise generated by a rotating device is dominated by the frequency of its rotation. Autocorrelation is the correlation of a signal with a time delayed version of itself, which can be used to identify the periodic signals, which in this case would be the sound generated by the disc. Therefore, autocorrelation of such an audio signal helps to determine the frequency and time period. The magnetic tachometer was designed to capture the magnetic field signal at an acquisition rate of 50 Hz and convert it into a frequency spectrum using Fast Fourier Transform (FFT) of 256 samples (Fig. S5). The peak frequency of the spectrum is then calculated, which corresponds to the rotational speed of the disc. To generate a periodic magnetic signal with the rotation of the disc, a small magnet was embedded in the disc.

A comparison of the smartphone-based tachometer (Magnetic and Acoustic) with a standard laser-based tachometer was made by measuring the rotational speed of the disc in the range of 0 to 3000 RPM. The results are shown in Fig. 3(b). It was observed that the magnetic tachometer works best for the rotational speeds from 0 to 1500 RPM and the acoustic tachometer works best for RPM greater than 1000. The limitation of magnetometer to measure higher RPM accurately can be attributed to the maximum sampling frequency of the magnetometer. The limitation of acoustic tachometer to measure at lower frequencies is because the microphones are not designed to pick up audio signal with very low frequencies. According to Nyquist Theorem, any periodic signal needs to be sampled at more than twice its highest frequency component for accurate measurement. Denoting sampling frequency as *f*_*sampling*_ and the maximum frequency of the signal as *f*_*max,signal*_, this can be written as follows:

1 $${f}_{sampling} \ge 2 \times {f}_{signal, max}$$

According to the manufacturer’s website, the sampling frequency of the magnetometer is 50 Hz for the phone used (Xiaomi Redmi Note 7) for this experiment, which implies, according to (1), that the maximum frequency the magnetometer can measure accurately will be below 25 Hz (1500 RPM).

### Microfluidic manipulation on the disc

**Fig. 4:**
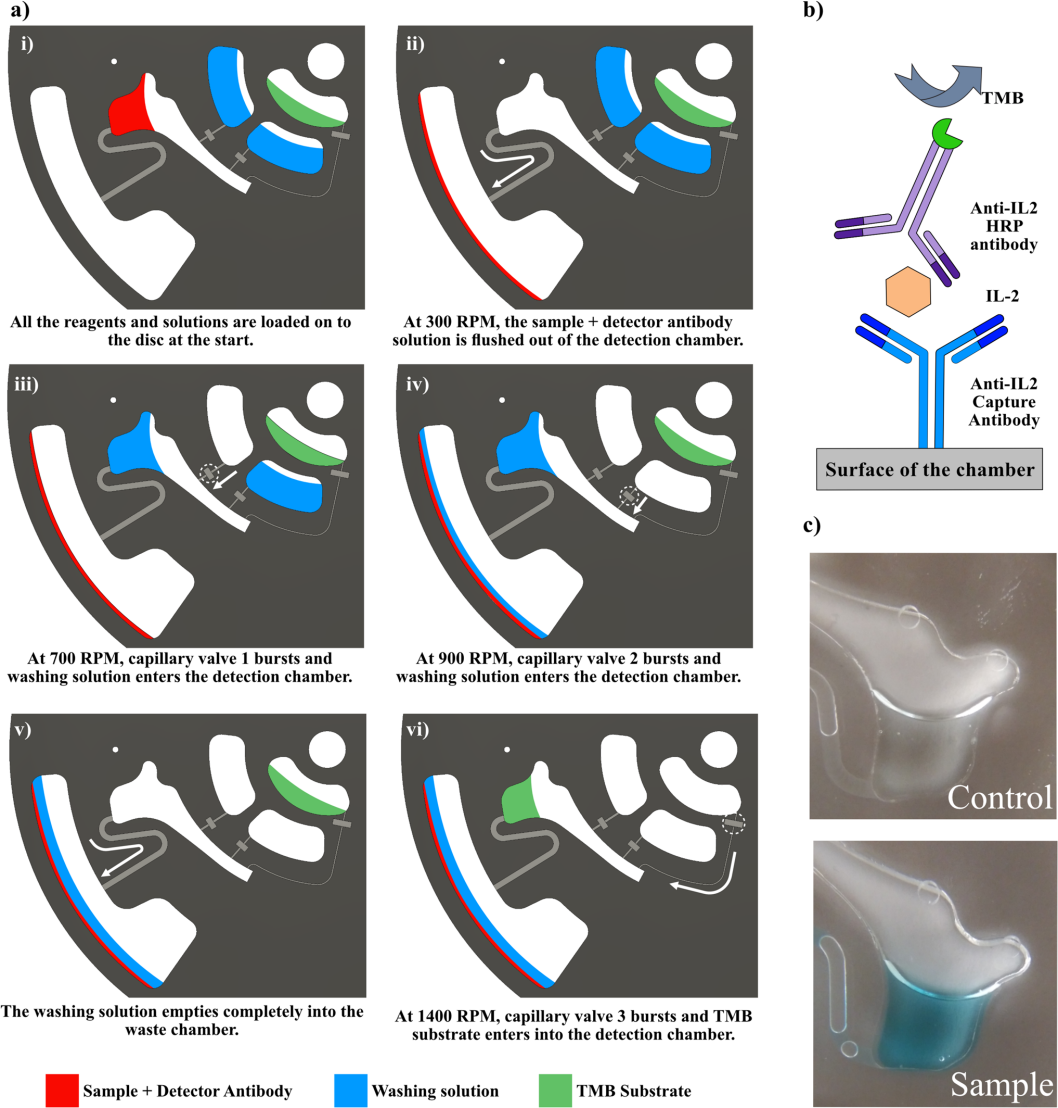
** a**) Schematics depicting stepwise microfluidic manipulation on the disc for automation of ELISA steps. Figures (i-vi) show the automated flow of sample, washing solutions and TMB substrate on the disc controlled by the speed of rotation. **b**) Schematic representation of the sandwich immunoassay for detection of IL-2. **c**) Result of the IL-2 sandwich immunoassay. Top picture shows the color development in the detection chamber with control (0 ng/mL) at the end of the IL-2 Assay. Bottom picture shows the color development in the detection chamber with the sample (200 ng/mL) at the end of the IL-2 Assay

Figure 4(a) shows a schematic description of the microfluidic manipulation on the disc. The disc uses a combination of channel geometries and different types of valves, namely capillary valve and siphon valve, to achieve an automated workflow. The fundamentals and working principle of a capillary valve and a siphon valve have been explained elsewhere (Cho et al. 2007; Duffy et al. 1999; Ducrée et al. 2007; Siegrist et al. 2009). Briefly, a capillary valve functions by stopping the flow of liquid by trapping the meniscus as it encounters a sudden expansion of the microchannel. The liquid stays trapped until the driving force exceeds the resisting capillary force and bursts the valve. A siphon valve consists of a chamber connected to a siphon channel that goes in the radially inward direction and then radially outwards below the chamber. While the disc is rotating at high speed, the centrifugal force keeps the liquid inside the chamber and the siphon channel. As the rotation speed is decreased, the capillary force overcomes the centrifugal force and pushes the liquid over the siphon crest. As the liquid moves down the siphon channel into the waste chamber, the sudden expansion acts as capillary valve. To burst this capillary valve, the rotational speed needs to be increased again, and as this happens it completely pumps the liquid out of the chamber into the waste.

Figure 4a (i) shows the start where sample solution containing the detector antibodies has been loaded into the detection chamber and washing solutions and TMB substrate in their respective chambers. As the disc is not rotating, the solution from the chamber creeps into the siphon channel and stops at the capillary valve at the entrance of waste chamber. In Fig. 4a (ii), the disc is rotating at 300 RPM and the sample solution is being emptied from the detection chamber into the waste chamber as it bursts the capillary valve. Figure 4a (iii) show the first washing step at 700 RPM when one of the capillary valve bursts and allows the washing solution to flow into the detection chamber and then through the siphon, into the waste chamber. Figure 4a (iv) shows the same procedure for the second washing solution where the capillary valve bursts at 900 RPM. Figure 4a (v) shows that both the washing solutions have been completely emptied from the detection chamber. Figure 4a(vi) shows that at 1400 RPM the TMB substrate flows into the detection chamber as capillary valve 3 bursts and then the rotation is stopped to incubate TMB for 3 min and allow the colorimetric signal to develop.

### Integration of ELISA steps

Fig. S6 shows experimental images of the stepwise workflow of the microfluidic disc that was used for conducting the sandwich immunoassay for detecting IL-2. The images were captured using a stroboscope and a high-speed camera. Figure 4(b) shows a schematic of the assay strategy used for detection of IL-2. The capture antibody is immobilized on the surface of the chamber through physical adsorption. This capture antibody binds to the IL-2 target and Anti-IL2 (HRP) antibody complex in the sample. For colorimetric detection, TMB substrate is used to generate a signal. To automate ELISA steps for the end user, it is assumed that the chamber has already been coated with capture antibody and the free sites on the surface of the chamber have been blocked. The end user starts by mixing the sample with HRP conjugated detector antibody and adds the solution to the detection chamber. From this point on, the remaining steps are automated as described in the previous section. Figure 4(c) shows the color development in the detection chamber with the control (0 ng/mL) and the sample (200 ng/mL) at the end of the IL-2 Assay. For quantification purposes, the solution from the detection chamber was collected and added to a well on 96-well plate having 70 μL of stop solution (2 M H_2_SO_4_) and the absorbance was measured in the spectrophotometer.

### IL-2 sandwich ELISA: Conventional 96-well plate vs centrifugal platform

**Fig. 5:**
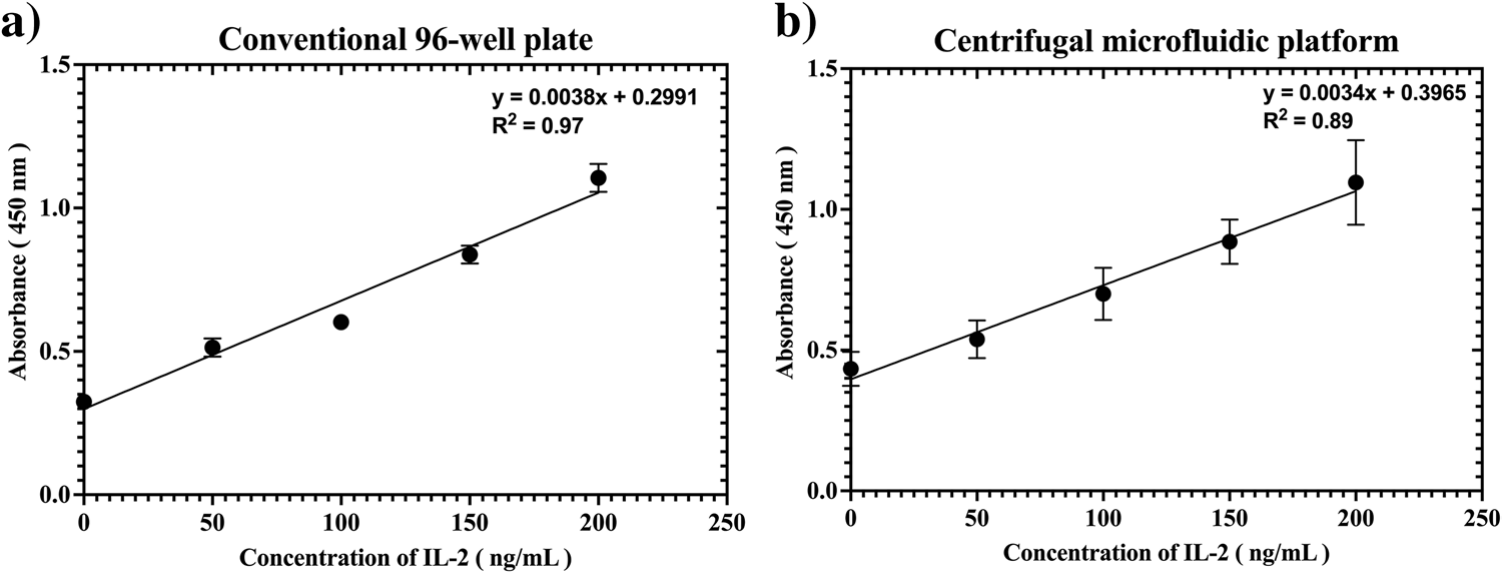
Speed control on the centrifugal microfluidic platform. **a**) Graph showing the relationship between voltage, current draw, and the rotational speed. **b**) Comparison between smartphone-based tachometer (magnetic and acoustic) and a standard laser-based tachometer. The error bars correspond to standard deviation of three measurements

To compare the performance of the platform with a conventional benchtop protocol, detection of IL-2 was carried out in 96-well plates. Same concentrations of the capture and detection antibody were used in both cases. Figure 5 compares the sandwich ELISA results on a 96-well plate and on the smartphone powered centrifugal microfluidic platform.

As shown in Fig. 5 there is a linear relationship between the colorimetric signal and antigen concentrations (0–200 ng/mL) in the 96-well plate. A linear signal was also observed for the sandwich ELISA on the centrifugal platform. Limit of detection (LOD) was calculated to be 16.27 ng/mL for the conventional 96-well plate and 65.17 ng/mL for the centrifugal disc. These results show that the platform can perform a multi-step ELISA. The relatively high LOD achieved in both cases can be attributed to the non-specific binding of the detection antibody to the walls of the wells or detection chamber, which leads to a high background signal. Moreover, the capture antibody was immobilised on the surface through passive adsorption which is driven by hydrophobic interactions and can result in denaturation, poor immobilization efficiency or improper orientation (Bai et al. 2006; Butler et al. 1992). Nonetheless, as compared to PMMA used for the fabrication of disc, the commercial 96-well plates are manufactured specially to facilitate a uniform binding of antibodies and low background signal (Mishra et al. 2018). Therefore, the adverse effect of physical adsorption of capture antibody and non-specific binding on the surface of the disc is expected to be more evident as compared to the 96-well plate. In order to minimize the non-specific binding on the disc, the surface of the detection chamber can be chemically modified to ensure a more uniform coating of the capture antibody by covalent linking or affinity based linkage to reactive groups on the surface (Davies et al. 1994). Another approach could be the use of agarose beads as solid phase for sandwich immunoassay as they have been reported to provide a performance similar to commercial ELISA kits (Pinto et al. 2021), and their combination with centrifugal microfluidics has been reported with a simplified colorimetric readout (Akhtar et al. 2023). However, it is beyond the scope of this work as the purpose of this assay was to demonstrate the automation of multi-step ELISA on the low-cost platform.

To conclude, integration and automation of multi-step ELISA was demonstrated using the platform with a performance similar to the manual liquid handling on conventional 96-well plate. The protocol involved manual steps for coating of capture antibody and the blocking of the chamber walls, but the discs are envisaged to be provided, similar to 96-well plates in an ELISA kit, with the capture antibody already coated on the surface of the detection chamber and the surface already blocked to prevent non-specific interactions. As a proof-of-concept, it was demonstrated that the platform can be readily adapted to detect other biomarkers using sandwich ELISA.

### Hematocrit level test

The measurement of hematocrit in blood is a common blood test used for medical diagnostic as a part of complete blood count (CBC) test (Riegger et al. 2007). Elevated levels of hematocrit are associated with heart diseases and disorders (Maslow et al. 2016; Harnett et al. 1995; Jin et al. 2015), whereas low levels can be indicative of anaemia or vitamin deficiencies (Thompson et al. 2016). In order to measure hematocrit level a simple structure was used that has been previously reported and allows for fast metering (Thompson et al. 2016). Briefly, the structure consists of a U-shaped channel with an inlet on one side and an overflow arm on the other side which leads into the waste chamber. The U-shaped channel facilitates sedimentation of red blood cells. Whereas the overflow arm combined with the waste chamber allows for accurate volume definition.

**Fig. 6:**
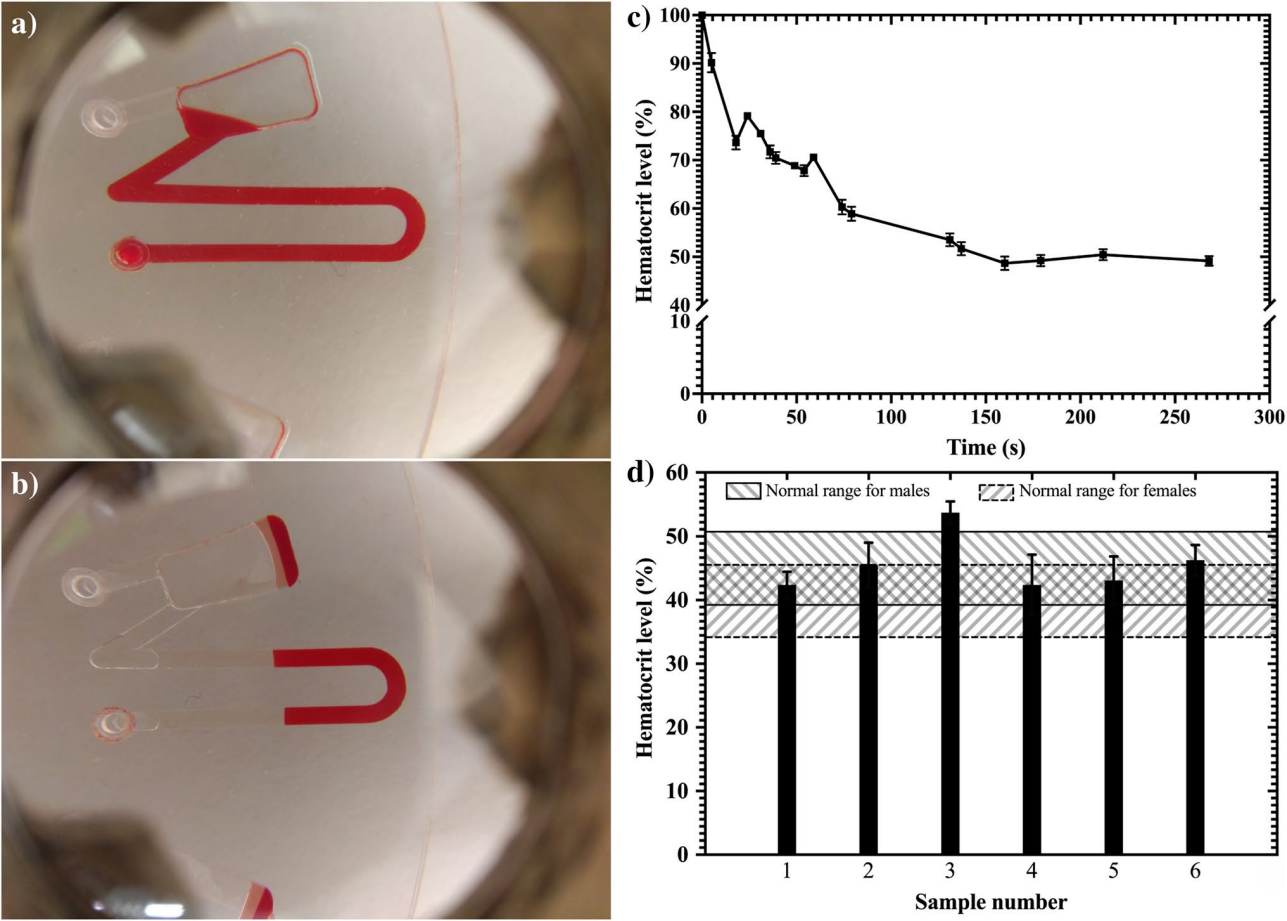
Hematocrit measurement using smartphone powered centrifugal microfluidic platform. **a**) fluidic structure filled with whole blood for measuring hematocrit level before centrifugation. **b**) fluidic structure filled with whole blood for measuring hematocrit level after centrifugation. **c**) the centrifugation time required for acquiring stable hematocrit level. **d**) The hematocrit levels measured for six random blood samples in triplicate using our centrifugal platform

After filling and running the test, the images generated from the cell phone were processed using a custom-written application in MATLAB, as described in our previous work (Banerjee et al. 2019). Briefly, images of the metered blood samples before and after blood plasma separation were captured and analysed. Figure 6(a) shows the microstructure filled with whole blood before spinning and Fig. 6(b) shows the hematocrit level when it reaches a final steady value. The hematocrit value changes rapidly in the beginning i.e., for the first 60 s and reaches a stable point 200 s onwards. These results are similar to those previously published by Thompson et al. (Thompson et al. 2016). Fig. S7 shows the binary threshold image and a histogram of the image generated for each of the existing pixel values. The images were converted to gray scale and the resulting number of darkest pixels in the image represents the hematocrit concentration i.e., the percentage of the total hematocrit in the U-shaped design. Figure 6(c) shows the mean time required for hematocrit level to reach a stable value. Figure 6(d) shows the results for hematocrit level measurement in blood for six different anonymous blood samples. For humans, the normal range of hematocrit values is between 30 to 50% i.e., it varies between females and males (Thompson et al. 2016). Different discs were used, and the measurements were repeated three times for six samples. For quantification, the image processing algorithm mentioned in section II-G was used and a standard deviation was calculated corresponding to each sample. Previously published data was used to ascertain whether the measured results were in the range of hematocrit level for normal samples. The clinically obtained hematocrit level data for both male and female donors is shown in Fig. 6(d). The figure shows that the hematocrit level of normal samples measured with the platform is in the range of hematocrit level in males and females (Thompson et al. 2016), except one of the samples. Therefore, it can be ascertained that the platform can either be used as a sample preparation method for assays that require blood plasma separation, or the blood plasma separation could be integrated on the disc to serve as the first step for a multi-step assay.

## Conclusions

The total cost, multi-functionality and access to electricity are some of the factors that limit the application of POC diagnostic systems in resource limited settings. A portable, low cost and robust diagnostic platform has been introduced here that is based on the combination of mobile phone and centrifugal microfluidics. It replaces the costly modules of centrifugal microfluidic platforms by using a low-cost housing made of cardboard, a motor, a speed controller, a lens and a mobile phone. The platform shows the potential of centrifugal microfluidics to carry out multi-step diagnostic assays in resource limited settings and remote areas. The wide availability of cardboard, the ease of assembly of the platform and the simplicity of the components used makes it a very attractive solution for testing in areas with minimal resources. The experimental results show that the electrical power stored in mobile phones typically generates adequate energy for conducting common assays on a centrifugal microfluidic device. As a proof of concept, colorimetric sandwich ELISA was carried out for detecting interleukin-2 on the platform. The platform was also used for measuring hematocrit level in whole blood by conducting plasma separation, metering, and analysing the results. The reported platform has certain limitations such as the power supplied by mobile phone battery can only be used with small DC motors, hence limiting to some extent the type of procedures it can carry out. The speed control mechanism used in the platform requires the user to constantly observe the speed in real time while changing it. We believe that these challenges can be overcome, and the overall usability of the platform be improved significantly by developing a mobile phone app that controls the platform through a microcontroller. Furthermore, the platform needs to be tested with biological samples. Building on our previous work (Akhtar et al. 2023), we envisage the integration of the plasma separation with the automated sandwich immunoassay for a sample-to-answer diagnostic platform. Overall, the results highlight the potential of the platform, its adaptability and indicate that it can be extended to perform various other diagnostic assays in resource limited settings.

## Supplementary Information

Below is the link to the electronic supplementary material.

Supplementary file1 (PDF 7194 KB)

Supplementary file2 (MP4 21508 KB)

Supplementary file3 (MP4 14778 KB)

## Data Availability

No datasets were generated or analysed during the current study.
