# Supplementary material for: Smartphone-driven centrifugal microfluidics for diagnostics in resource limited settings
Source: Biomed Microdevices. 2024 Oct 26;26(4):43. doi: 10.1007/s10544-024-00726-x (PMC11512838; doi:10.1007/s10544-024-00726-x)
Supplement: Supplementary file 1 — Supplementary file1 (PDF 7194 KB) [file 10544_2024_726_MOESM1_ESM.pdf]

Supplementary Information

Smartphone-driven centrifugal microfluidics for diagnostics in resource limited settings

*Noa Lapins [a, *]; Ahmad S. Akhtar [a, *]; Indradumna Banerjee [a]; Amin Kazemzadeh [a]; Inês F. Pinto [a]; Aman Russom [a,b, †]*

[a] KTH Royal Institute of Technology, Division of Nanobiotechnology, Department of Protein Science, Science for Life Laboratory, Solna, Sweden

[b]AIMES – Center for the Advancement of Integrated Medical and Engineering Sciences at Karolinska Institutet and KTH Royal Institute of Technology, Stockholm, Sweden

[*] These authors contributed equally

[†] Contact authors: aman@kth.se

Table of Contents

Fig. S1: Folding instructions for the cardboard housing......2
Fig. S2: Circuit schematic for powering the motor.......2
Section S1: Fabrication of the centrifugal disc......3
Fig. S3: Schematic representation of the disc design......3
Fig. S4: Flow chart explaining the customized algorithm of acoustic tachometer......4
Table S1: Raw data for comparison of acoustic tachometry to standard laser-based tachometry......4
Fig. S5: Flow chart explaining the customized algorithm of magnetic tachometer......5
Table S2: Raw data for comparison of magnetic tachometry to standard laser-based tachometry......5
Fig. S6:Images from stroboscope setup showing step-wise flow of liquid......6
Fig. S7: Hematocrit level measurement using image processing......7
Movie S1......7
Movie S2......7
Table S3. List of Abbreviations......8

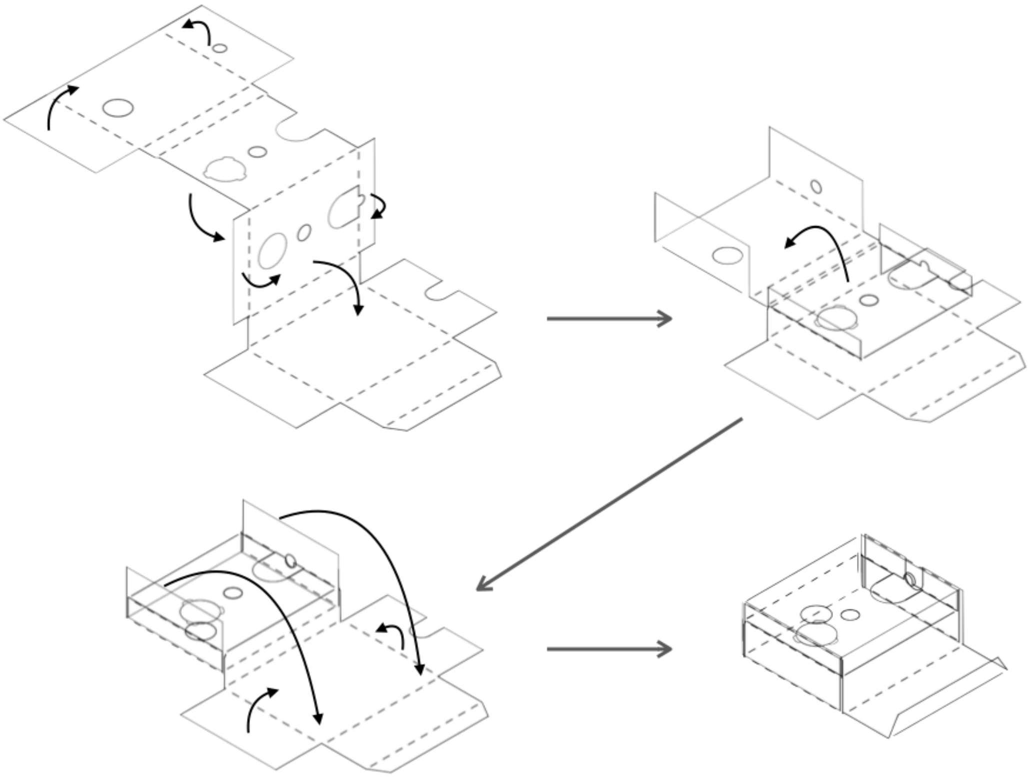

Fig. S1: Folding instructions for the cardboard housing. The cardboard is partially punctured, indicated by the dotted lines, that allow the end-user to assemble the housing according to instructions.

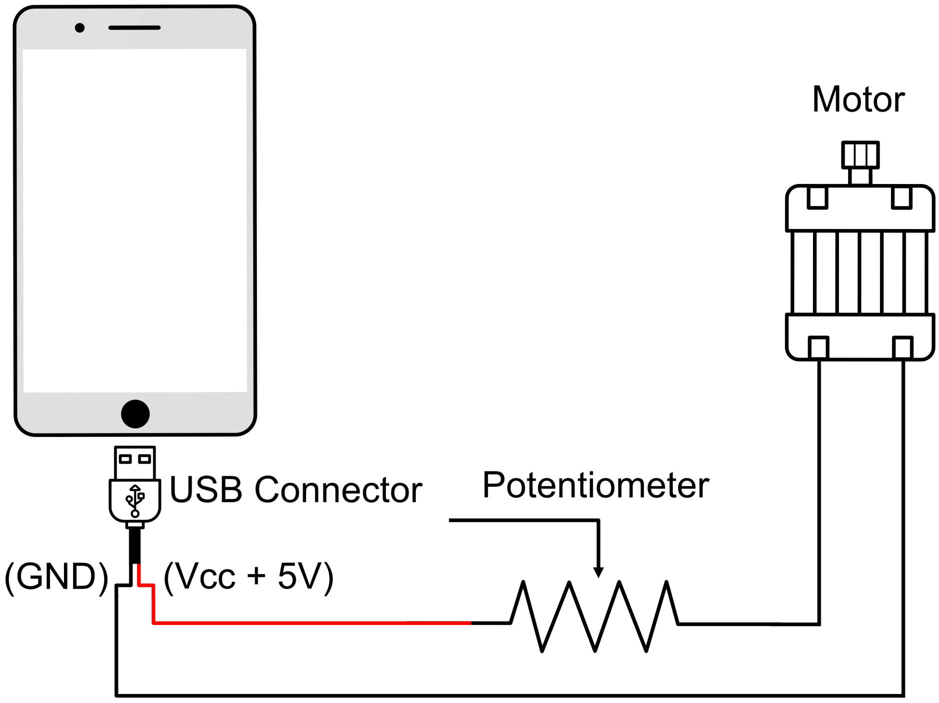

Fig. S2: Circuit schematic for powering the motor using the electrical energy of the mobile phone. The potentiometer is used for speed control.

Section S1: Fabrication of the centrifugal disc

For integration of assay steps on the centrifugal microfluidic disc, the disc design is as shown in Figure S3. The diameter of the disc is 106 mm and the diameter of the central mounting hole is 6.2 mm. The design consists of five separate chambers (i) Detection chamber for sample with pre-added detector antibody, (ii) and (iii) for washing solutions, (iv) for TMB substrate and (v) waste chamber. The detection chamber is connected to the inlet chambers and to the waste chamber through channels of different dimensions and valves.

The detection chamber is connected to the washing solution chamber I and II through channels having dimensions of 400 × 400 µm and 300 × 300 µm respectively. The channels connecting to washing solution I and II have a capillary valve at a radial distance of 23 mm and 21.5 mm respectively. The chamber with TMB substrate connects to the detection chamber through a a 200 × 200 µm channel with a capillary valve at a radial distance of 11.5 mm. The detection chamber connects with the waste chamber through a siphon valve.

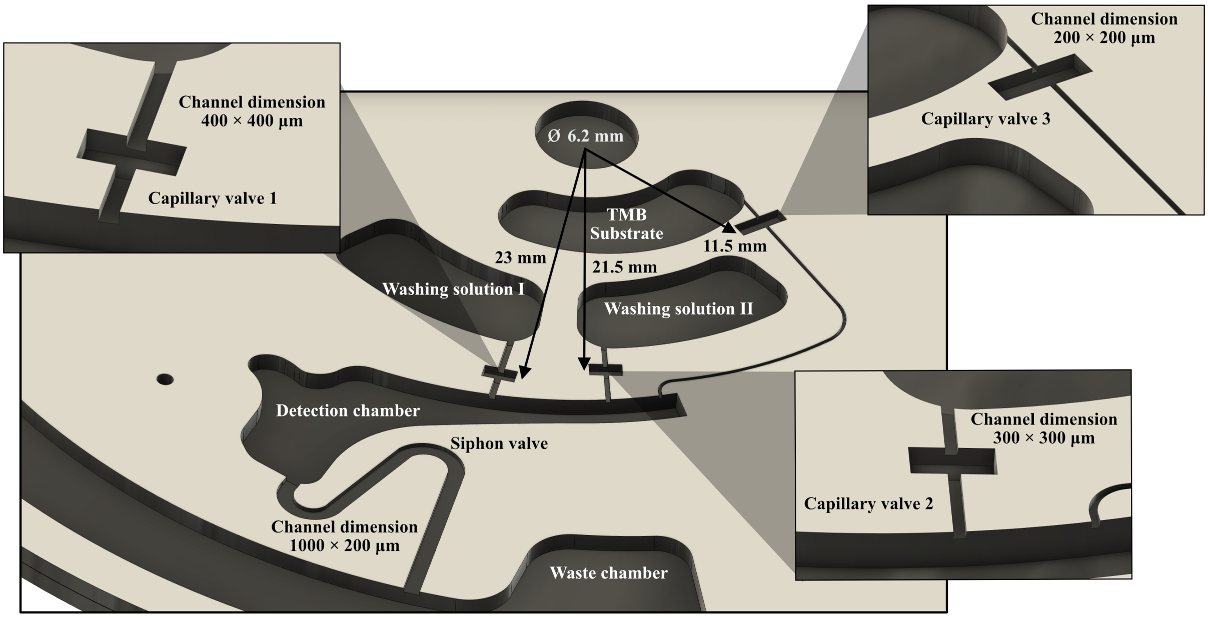

Fig. S3: Schematic representation of the disc design with channel dimensions and a zoomed in view of the different valves.

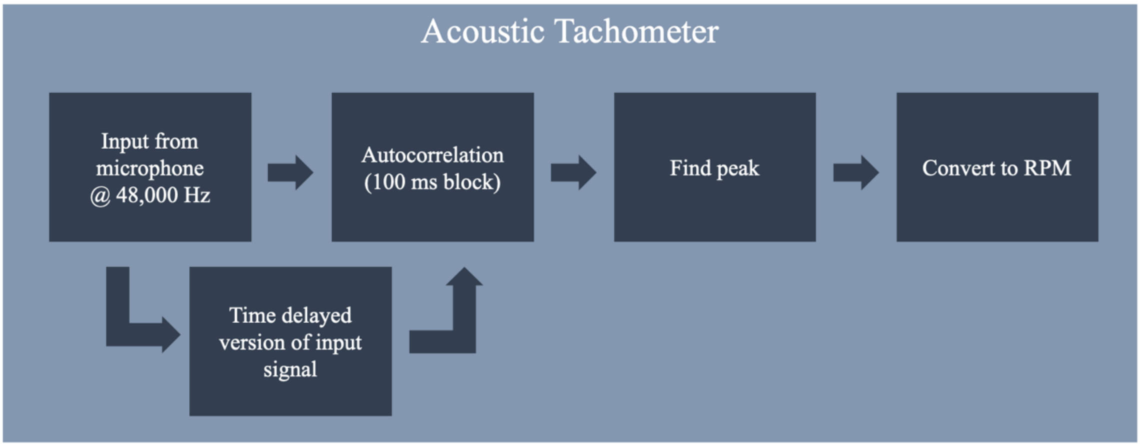

Fig. S4: Flow chart explaining the customized algorithm of acoustic tachometer in the phyphox app.

**Laser based tachometry (Standard)**	**Acoustic tachometry using Phyphox App**	
	1st Measurement	2nd Measurement
**880**	860.54991	904.050235
**1115**	1095.24534	1110.02313
**1308**	1307.03586	1306.44283
**1560**	1579.48437	1576.88938
**1691**	1691.77438	1689.78873
**1819**	1823.55921	1825.87191
**2044**	2046.48188	2050.8547
**2197**	2191.3242	2189.65779
**2448**	2448.46939	2448.46939
**2550**	2552.65957	2577.79767
**2694**	2706.20301	2711.29944
**2865**	2865.07463	2865.07502
**2961**	2962.34568	2962.34568
**3075**	3069.72281	3072.99893

Table S1: Raw data for comparison of acoustic tachometry to standard laser-based tachometry. The two values of acoustic tachometry represent two measurements using the app.

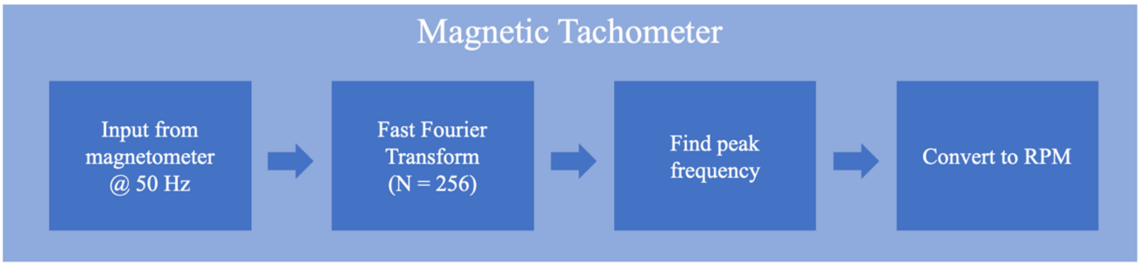

Fig. S5: Flow chart explaining the customized algorithm of magnetic tachometer in the phyphox app.

**Laser based tachometry (Standard)**	**Magnetic tachometry using Phyphox app**	
	1st Measurement	2nd Measurement
**303**	304.854791	304.854904
**460**	445.557319	445.556865
**780**	773.86287	797.309035
**960**	961.465683	961.465489
**1136**	1125.61803	1149.0725
**1290**	1384.5882	1384.25016
**1360**	1291.66679	1314.82914
**1479**	1478.07973	1383.8954

Table S2: Raw data for comparison of magnetic tachometry to standard laser-based tachometry. The two values of magnetic tachometry represent two measurements using the app.

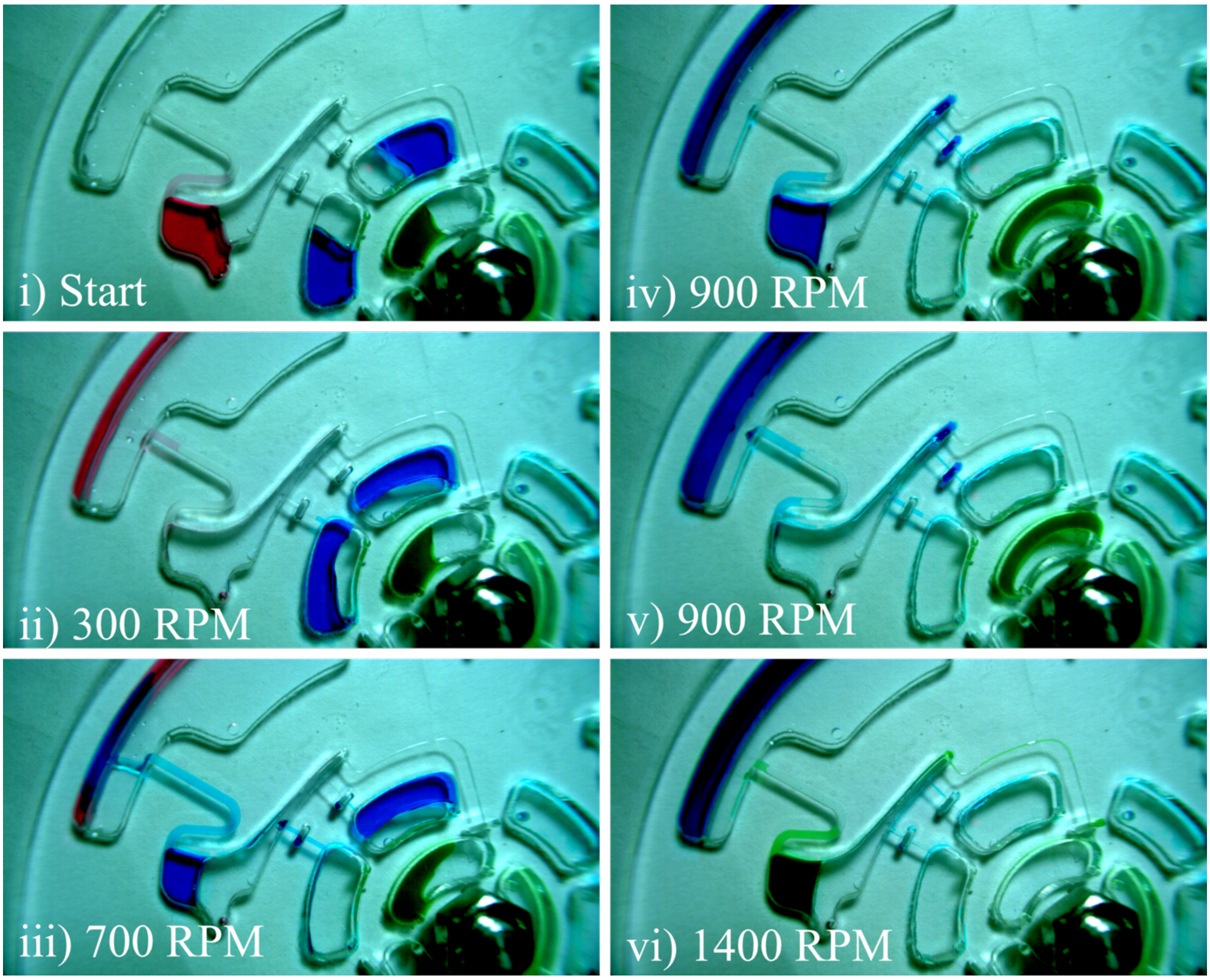

Fig. S6: To visualize each step of the assay, the disc design is shown with all chambers filled with food dye in DI water. First image (i) represents loading of sample and detection antibody, (ii) then at 300 RPM the detection chamber is emptied. (iii) and (iv) At 700 RPM and 900 RPM first and second washing solution are flushed into the detection chamber and through the siphon to the waste chamber (v), sequentially. (vi)At 1400 RPM the substrate fills the detection chamber where the signal is developed.

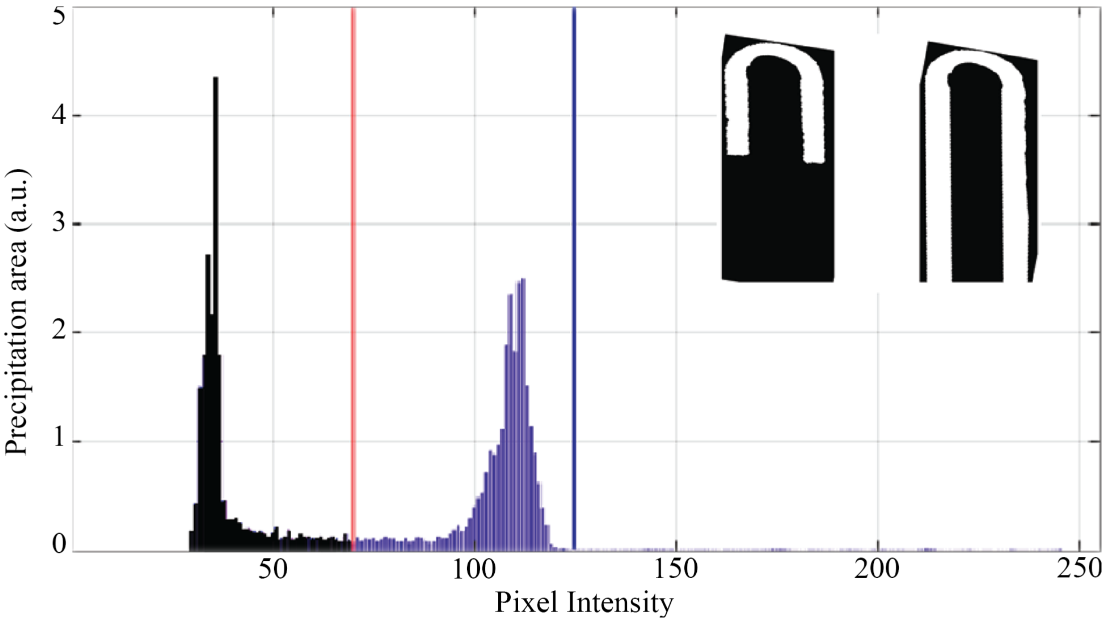

Fig. S7: Binary threshold image and histogram of the image of the channel for hematocrit level measurement using image processing, as previously described by our group (Banerjee et al., 2019).

Supporting movie captions

Movie S1

The supplementary video demonstrates the assembly of cardboard housing by inserting all the components and folding the cardboard according to the folding instructions.

Movie S2

The supplementary video S2 shows the steps for imaging of a channel on the disc. It involves opening the flap of the platform to access the disc and moving it to the desired channel. Then then flap is closed, and the disc is ready to be imaged. It also demonstrates the use of camera flash of the phone to illuminate the chamber for imaging.

References

BANERJEE, I., ARALAGUPPE, S. G., LAPINS, N., ZHANG, W., KAZEMZADEH, A., SöNNERBORG, A., NEOGI, U. & RUSSOM, A. 2019. Microfluidic centrifugation assisted precipitation based DNA quantification. *Lab on a Chip,* 19**,** 1657-1664.

Table S3. List of Abbreviations

POC	*Point-Of-Care*
RPM	*Revolutions Per Minute*
IL-2	*Interleukin-2*
LOD	*Limit Of Detection*
REASSURED	*Real time connectivity, Ease of sample collection, Affordable, Sensitive, Specific, User friendly, Rapid and robust, Equipment free and Deliverable to end users*
RLS	*Resource Limited Settings*
COVID-19	*Coronavirus disease 2019*
WHO	*World Health Organization*
HIV	*Human Immunodeficiency Virus*
NAAT	*Nucleic Acid Amplification Test*
PC	*Personal Computer*
LAMP	*Loop Mediated Isothermal Amplification*
OTG	*On-The-Go*
DC	*Direct Current*
PMMA	*Polymethylmethacrylate*
ELISA	*Enzyme Linked Immunosorbent Assay*
TMB	*Tetramethylbenzidine*
PBS	*Phosphate Buffered Saline*
HRP	*Horse Radish Peroxidase*
BSA	*Bovine Serum Albumin*
EDTA	*Ethylenediaminetetraacetic Acid*
CBC	*Complete Blood Count*
